# The Thermal Performance Analysis of an Al_2_O_3_-Water Nanofluid Flow in a Composite Microchannel

**DOI:** 10.3390/nano12213821

**Published:** 2022-10-28

**Authors:** Mirza Farrukh Baig, Gooi Mee Chen, Chih Ping Tso

**Affiliations:** Centre for Advanced Mechanical and Green Technology, Faculty of Engineering and Technology, Multimedia University, Bukit Beruang, Melaka 75450, Malaysia

**Keywords:** viscous dissipation, porous medium, forced convection, nanofluid, local thermal non-equilibrium

## Abstract

Partial filling of porous medium insert in a channel alleviates the tremendous pressure drop associated with a porous medium saturated channel, and enhances heat transfer at an optimum fraction of porous medium filling. This study pioneered an investigation into the viscous dissipative forced convective heat transfer in a parallel-plate channel, partially occupied with a porous medium at the core, under local thermal non-equilibrium condition. Solving the thermal energy equation along the Darcy–Brinkman equation, new exact temperature fields and Nusselt number are presented under symmetrical isoflux thermal boundary condition. Noteworthy is the heat flux bifurcation at the interface between the clear fluid and porous medium driven by viscous dissipation, in cases where the combined hydrodynamic resistance to fluid flow and thermal resistance to fluid conduction is considerable in low Darcy number porous medium insert. However, viscous dissipation does not affect the qualitative variation of the Nusselt number with the fraction of porous medium filling. By using Al_2_O_3_-Water nanofluid as the working fluid in a uniformly heated microchannel, partially filled with an optimum volume fraction of porous medium, the heat transfer coefficient improves as compared to utilizing water. The accompanied viscous dissipation however has a more adverse impact on the heat transfer coefficient of nanofluids with an increasing Reynolds number.

## 1. Introduction

Forced convection heat transfer in porous medium is encountered in broad engineering applications, such as heat exchangers, oil recovery, electronics cooling, heat pipes, catalytic reactors, fuel cells, thermal insulation, geothermal systems, and many more [1]. To cut down the tremendous pressure drop required to drive a flow through a channel saturated with porous medium, partial filling of porous medium becomes an alternative to augment convection heat transfer while maintaining the pumping cost at an acceptable level [2,3,4].

Studies on a channel partially filled with porous medium are less common than ones which are fully filled. Past studies in references [3,5,6,7,8,9,10,11,12,13,14,15] developed the velocity and temperature distributions based on the local thermal equilibrium (LTE) assumption in a channel partially filled with porous medium. The interests span various configurations of porous medium insert [3,5,6,7,8,9,10,11,12,13,14,15], laminar and turbulent flow [6,7], thermal dispersion effect [9], effect of moving boundary condition [10], and pulsating flow [15]. The fluid flow and heat transfer at the interface region was analyzed for three different cases, namely two different porous medium, a fluid region, and a porous medium, and lastly, an impermeable medium and a porous medium [5]. The study derived velocity and temperature distribution for all three interface conditions, and found great agreement between their analytical and numerical solutions, by using the empirical hypothesis [8]. The non-Darcy effects were numerically studied on the fully developed forced convection in a parallel plate channel partially filled with a porous medium, by assuming Darcy–Brinkman–Forchheimer model in a porous medium [9]. The study indicated that convective heat transfer enhances the thermal dispersion effect, more significantly at smaller values of thickness ratio for the fluid layer to porous medium layer. An investigation on a Couette flow in a composite channel partially filled with a porous medium, was carried out by adopting the Brinkman–Forchheimer–extended Darcy equation, for the porous medium region [10]. The study obtained the velocity and temperature distributions by using the stress jump condition at the interface region [11] and showed that Nusselt number depends on the size of the gap between a porous medium and moving plate; Nusselt number increases with a decreasing gap size and Darcy number $\left(Da\right)$. A partially filled channel with porous medium placed at the core, imposed with symmetrical boundary heat flux condition, was analyzed both analytically and numerically in another study [12]. The use of porous layer for heat transfer enhancement and the accompanying pressure drop was evaluated. The study showed that the overall performance is more strongly affected by Da, but not so much by the thermal conductivity ratio of porous medium to fluid layer, as the ratio increases beyond two. Subsequently, a theoretical analysis was conducted on the heat transfer and fluid flow of a channel, with a porous medium inserted adjacent to the wall, assuming uniform heat flux thermal boundary condition [13]. The study perceived a good agreement between the analytical and numerical results, and showed that the maximum overall Nusselt number based on the definition of mean thermal conductivity of porous medium and clear fluid is attainable for a thermal conductivity ratio of one. Notably, the location of porous medium insert in a circular pipe influenced heat transfer rates substantially [14]. A study on the heat transfer for a pulsating flow in a circular pipe partially filled with porous medium was tackled numerically [15]. The study analyzed the effect of different parameters on convective heat transfer and determined the optimal thickness of the porous layer.

LTE assumption in the aforementioned studies is valid when there is a negligible temperature difference between solid and fluid phases. Local thermal non-equilibrium (LTNE) or two-equation model in a porous medium reflects the fluid and solid temperatures more accurately. However, investigations on the enhancement of heat transfer in a channel partially filled with porous medium under LTNE conditions remain relatively scarce. One area of interest lies in the modelling of temperature and heat flux at the interface between the porous medium and clear fluid. For a uniform heat flux boundary condition, two approaches were proposed to model the thermal boundary condition at the interface [16]. In the first approach, depending upon the effective conductivities and temperature gradient of solid and fluid phases, the total heat flux is divided between two phases, whereas an equal amount of heat flux received by individual phases at the boundary is modelled in the second approach. In another study, a jump condition was introduced at the interface between a porous medium and a homogenous fluid [17]. The study used the volume-average technique for thermal energy equations in solid and fluid phases and showed that a jump condition contains an excess in heat flux at an interface. A systematic analysis was presented to model different interfacial momentum and thermal boundary conditions between a porous medium and a fluid layer [18]. Variances among different models were compared and reviewed, revealing that for most of the practical applications, the variance in different models has the least impact on temperature and Nusselt number distributions. Another study adopted the two aforementioned approaches [16] to model heat transfer in porous medium, and termed the varied heat flux, and uniform heat flux interface models as model A, and model B respectively, in the channels partly occupied by porous medium [19]. Under LTNE condition, and with model A applied at the interface, the study obtained the exact temperature field for a parallel plate partially occupied by porous insert [4]. Nusselt number is affected by three conditions, namely channelling effect, fluid conduction from the interface, and solid conduction and internal heat exchange in a porous medium. The study also highlighted that an increased thickness of porous medium insert amplified the error in an LTE model and suggested the optimum fraction of porous material to enhance the heat transfer to be 0.8 under reasonable pressure drop. Amid growing interest to examine the validity of interface boundary conditions between two regions, a study reported the restrictions on the validity of thermal boundary conditions at the interface and presented the largest Nusselt number for model A [20]. The study further explored the heat flux bifurcation inside the composite channel by considering the inertial and thermal dispersion effects, and applying three different interfacial thermal conditions [21]. The study discerned the heat flux bifurcation phenomenon at the porous-fluid interface when the temperatures of the solid and fluid phases are not equal, while the inertial terms show weaker effects when the thermal dispersion effect is included and vice versa. The exact solutions were obtained for both fluid and solid phases by inserting the metallic foams on both sides of the tube under the LTNE model [22]. In order to predict the effectiveness of inserting porous material into the tube, an analytical investigation was performed on forced convection in a heated tube with a porous medium core, and a tube with a porous medium insert at the side, adopting model A as a thermal boundary condition under the LTE, and LTNE conditions [23]. The study took into account of thermal dispersion effects and found that the heat transfer performance of the tube with a porous medium core is higher in a lower range of pumping power than that of the tube with porous medium layer at the side, while the latter’s performance exceeds the former in a higher range of pumping power. More recently, the variations between the resulting temperature distribution and the Nusselt number for model A and model B were analyzed [24,25]. An analytical solution was developed to predict the validity of the LTE model [24]. Although a higher Nusselt number was obtained for model A, the heat transfer performance depends less on the interface model and Da when the volume fraction of porous medium is less than 0.9. An analytical study on a partially filled channel under LTNE condition, subjected to a constant heat flux, with the porous insert attached to both sides of channel walls [26], was subsequently looked into. The study adopted the stress jump and continuity condition at the interface and examined the effects of stress jump coefficient on the fluid flow and heat transfer using model A. It predicted an optimum volume fraction of 0.2 for clear fluid region in order to enhance the heat transfer. The Nusselt number depends more strongly on the thermal conductivity ratio than the porous medium fraction. The studies [4,22,23,26,27,28,29,30] showed that the more commonly used model A leads to a higher Nusselt number.

Viscous dissipation is a heat source owing to work done by viscous stresses. The effects of viscous dissipation were neglected in most analyses of forced convection in a channel partially filled with porous medium, exposed to LTNE conditions [4,20,23,24,26,27,31]. Studies [29,32] show that the internal heat source alters the temperature, Nusselt number, and entropy generation of the system significantly in either the solid or fluid phases in porous medium. With viscous dissipation incorporated in the energy equations, the mathematical model examined the heat transfer and entropy generation for a partially filled channel, subjected to constant heat flux at one wall, and an adiabatic condition on the other wall using the LTNE model [33]. The study reported the bifurcation of heat transfer and entropy generation as well as the possibility of having a temperature higher than that of the heated wall. A new analytical solution was next presented for a composite channel partly occupied by a porous material next to the wall [34]. The work highlighted heat flux bifurcation near the interface region and a more dominant viscous dissipation effect in the clear fluid region, intensified with an increasing Da. In another study, internal heat generation effects, within the fluid and solid phases of a porous medium under LTNE condition, for models A and B were analysed for a partially filled channel, along with the phenomenon of heat flux bifurcation at the porous-fluid interface [35]. The first and second law analyses were undertaken in a microreactor partially filled with porous medium insert, with internal heat generation [36]. Imposing asymmetrical temperatures at the boundary surface and assuming a thick outer wall, internal heat generations was found to significantly affect the system. In another study, three viscous dissipation models were studied, namely, the Darcy model, the form drag model, and the clear fluid compatible model, for a channel incorporated with a porous insert next to the wall [37]. The study presented an analytical solution, without involving the convection term, for the energy equation. The temperatures and heat transfer coefficients are affected more significantly by viscous dissipation with an increasing *Da*. The best heat transfer enhancement is achieved in the fluid-compatible viscous dissipation model. For both LTE and LTNE conditions, a recent study assessed the effect of internal heat sources on the thermal performance and reported that an increasing thickness of porous insert causes singularities in Nusselt number [38]. The study is however restricted to uniform heat sources in fluid and solid phases.

To this end, existing literature points to the fact that the LTNE model is able to present a more comprehensive temperature field, while viscous dissipation influences the temperature field more significantly in cases of large Da besides imposing heat flux bifurcations at the interface in a channel partially filled with porous medium adjacent to the wall. The configuration of the composite wall has also been highlighted to have an effect on the thermal performance. To the best of the authors’ knowledge, the effects of viscous dissipation on the thermal aspects of a composite channel, with a porous insert at its core, has not been investigated under LTNE condition despite its rather common configuration in a composite channel. This study hence intends to bridge the gap by focusing on the arising heat transfer characteristics manifested by viscous dissipation in the composite channel, uniformly heated at the boundaries. The results are subsequently applied to a composite microchannel, with Al_2_O_3_-Water nanofluid as the coolant.

## 2. Problem Formulation

**Figure 1:**
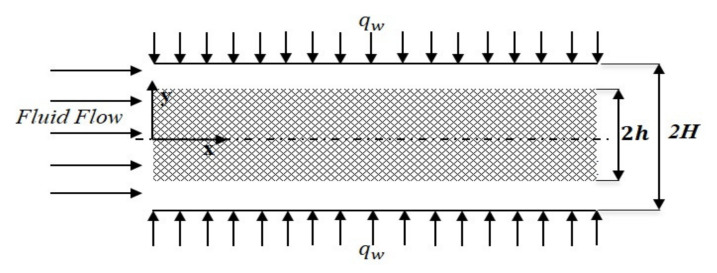
Schematic diagram of the problem.

This problem is formulated for forced convective heat transfer in a parallel plate channel, partially filled with porous medium at the core, as shown in Figure 1. The plates are separated by a distance 2H, with a porous medium of thickness 2h inserted at the core of the channel, subjected to isoflux thermal boundary condition. The flow is assumed to be hydrodynamically fully developed, steady, and laminar within the channel, while LTNE is assumed in the porous medium. Due to the symmetry of the problem, only half of the channel is solved in this study.

### 2.1. Problem Formulation

The velocity profiles presented in [24] are adopted in this study, obtained by solving the Navier Stokes equation and Brinkman-extended Darcy equation, in the clear fluid and porous medium, respectively. Interface continuity is assumed for the velocity and shear stress. The velocity distribution [24] is given as

(1) $$U_{c}=-\frac{Y^{2}}{2}+A_{1}Y+A_{2}$$

(2) $$U_{p}=A_{3}\cosh\left(A_{0}Y\right)+Da\;$$

where $A_{0}-A_{3}$ are the constants listed in Appendix A.

### 2.2. Problem Formulation

The governing thermal energy equations for the respective clear fluid, and LTNE porous medium, are [34]:

Clear fluid:

(3) $$k_{f}\frac{\partial^{2}T_{c}}{\partial y^{2}}+\mu_{f}{\left(\frac{du_{c}}{dy}\right)}^{2}=\rho_{f}c_{p,f}u_{c}\frac{\partial T_{c}\;}{\partial x}\;\;\;$$

Porous medium:

(4) $$k_{s,eff}\frac{\partial^{2}T_{s}}{\partial y^{2}}-h_{i}a_{i}\left(T_{s}-T_{f}\right)=0$$

(5) $$k_{f,eff}\frac{\partial^{2}T_{f}}{\partial y^{2}}+h_{i}a_{i}\left(T_{s}-T_{f}\right)+\frac{\mu_{f}}{K}u_{p}{}^{2}+\mu_{eff}{\left(\frac{du_{p}}{dy}\right)}^{2}=\rho_{f}c_{p,f}u_{p}\frac{\partial T_{f}\;}{\partial x}$$

where in the thermal energy for fluid in porous medium, Equation (5), a clear fluid compatible model [39] for viscous dissipation is incorporated.

In this study, the temperature at the interface between the clear fluid region and the porous medium is assumed to be constant. Based on the difference in effective thermal conductivities and temperature gradients between the two phases in a porous medium, heat flux is divided between clear fluid and porous medium [16].

The corresponding thermal boundary conditions are [16]

(6) $$y=0,\frac{\partial T_{f}}{\partial y}=0,\frac{\partial T_{s}}{\partial y}=0$$

(7) $$y=h,\;\;\;\;\;\;T_{f}=T_{s}=T_{c},\;\;\;\;\;\;k_{f,eff}\;\frac{\partial T_{f}}{\partial y}+k_{s,eff}\;\frac{\partial T_{s}}{\partial y}=k_{f}\;\frac{\partial T_{c}}{\partial y}=q_{int}$$

(8) $$y=H,\;\;\;\;\;\;k_{f}\frac{\partial T_{c}}{\partial y}=q_{w}$$

For a fully developed condition subjected to constant heat flux, $\frac{\partial T}{\partial x}=\frac{\partial T_{f}}{\partial x}=\frac{\partial T_{c}}{\partial x}=\frac{\partial T_{m}}{\partial x}=\mathrm{constant}\;$, and is computed from

(9) $$\frac{\partial T}{\partial x}=\frac{1}{\rho_{f}c_{p,f}u_{m}H}\left[q_{w}+\frac{\mu_{f}}{K}\overset{h}{\underset{0}{\int }}u_{p}{}^{2}dy+\frac{\mu_{f}}{\epsilon }\overset{h}{\underset{0}{\int }}{\left(\frac{du_{p}}{dy}\right)}^{2}dy+\mu_{f}\overset{H}{\underset{h}{\int }}{\left(\frac{du_{c}}{dy}\right)}^{2}dy\right]\;\;\;\;\;$$

where $u_{m}=\frac{1}{H}\left(\overset{h}{\underset{0}{\int }}u_{p}dy+\overset{H}{\underset{h}{\int }}u_{c}dy\right)$.

By introducing

(10) $$\theta =\frac{k_{s,eff}\left(T-T_{int}\right)}{q_{w}H},\;Bi=\frac{h_{i}a_{i}H^{2}}{k_{s,eff}},\;\kappa =\frac{k_{f,eff}}{k_{s,eff}},Br=\frac{\mu_{f}u_{m}{}^{2}H}{q_{w}K},\gamma =\frac{q_{int}}{q_{w}},\epsilon =\frac{k_{f,eff}}{k_{f}}$$

The resulting non-dimensional equations are

(11) $$\frac{\kappa }{\epsilon }\frac{\partial^{2}\theta_{c}}{\partial Y^{2}}=\frac{U_{c}\omega }{U_{m}}-\frac{BrDa}{U_{m}{}^{2}}{\left(\frac{dU_{c}}{dY}\right)}^{2}$$

(12) $$\kappa \frac{\partial^{2}\theta_{f}}{\partial Y^{2}}+Bi\left(\theta_{s}-\theta_{f}\right)=\frac{U_{p}\omega }{U_{m}}-\frac{Br}{U_{m}{}^{2}}U_{p}{}^{2}-\frac{BrDa}{U_{m}{}^{2}\epsilon }{\left(\frac{dU_{p}}{dY}\right)}^{2}$$

(13) $$\frac{\partial^{2}\theta_{s}}{\partial Y^{2}}-Bi\left(\theta_{s}-\theta_{f}\right)=0$$

where

(14) $$\omega =1+\frac{Br}{U_{m}{}^{2}}\overset{\eta }{\underset{0}{\int }}U_{p}{}^{2}dY+\frac{BrDa}{U_{m}{}^{2}\epsilon }\overset{\eta }{\underset{0}{\int }}{\left(\frac{dU_{p}}{dY}\right)}^{2}dY+\frac{BrDa}{U_{m}{}^{2}}\overset{1}{\underset{\eta }{\int }}{\left(\frac{dU_{c}}{dy}\right)}^{2}dY$$

The corresponding thermal boundary conditions are

(15) $$Y=0,\;\;\;\;\;\;\frac{\partial \theta_{f}}{\partial Y}=0,\;\;\;\;\;\;\frac{\partial \theta_{s}}{\partial Y}=0$$

(16) $$Y=\eta ,\;\;\;\;\;\;\theta_{f}=\theta_{s}=\theta_{c}=0,\;\;\;\;\;\;\frac{\kappa }{\epsilon }\frac{\partial \theta_{c}}{\partial Y}=\kappa \frac{\partial \theta_{f}}{\partial Y}+\frac{\partial \theta_{s}}{\partial Y}=\gamma$$

(17) $$Y=1,\;\;\;\;\;\;\frac{\kappa }{\epsilon }\frac{\partial \theta_{c}}{\partial Y}=1.\;$$

The resulting Equations (18) and (19) are obtained are after some manipulations to decouple Equations (12) and (13).

$$\frac{\partial^{4}\theta_{f}}{\partial Y^{4}}-\frac{Bi\left(1+\kappa \right)}{\kappa }\frac{\partial^{2}\theta_{f}}{\partial Y^{2}}=\frac{U_{p}\omega Bi}{\kappa U_{m}}-\frac{\omega }{\kappa U_{m}}\frac{d^{2}U_{p}}{dY^{2}}-\frac{BrBi}{\kappa U_{m}{}^{2}}\left[U_{p}{}^{2}+\frac{Da}{\epsilon }{\left(\frac{dU_{p}}{dY}\right)}^{2}\right]$$

(18) $$+\frac{Br}{\kappa U_{m}{}^{2}}\left[\frac{d^{2}U_{p}{}^{2}}{dY^{2}}+\frac{Da}{\epsilon }\;\frac{d^{2}{\left(dU_{p}/dY\right)}^{2}}{dY^{2}}\right]$$

(19) $$\frac{\partial^{4}\theta_{s}}{\partial Y^{4}}-\frac{Bi\left(1+\kappa \right)}{\kappa }\frac{\partial^{2}\theta_{s}}{\partial Y^{2}}=\frac{U_{p}\omega Bi}{\kappa U_{m}}-\frac{BrBi}{\kappa U_{m}{}^{2}}\left[U_{p}{}^{2}+\frac{Da}{\epsilon }{\left(\frac{dU_{p}}{dY}\right)}^{2}\right]$$

To solve Equations (18) and (19), additional thermal boundary conditions are derived from Equations (12) an (13), giving

(20) $$Y=0,\;\;\;\;\;\;\frac{\partial^{3}\theta_{f}}{\partial Y^{3}}=0$$

(21) $$Y=0,\;\;\;\;\;\;\frac{\partial^{3}\theta_{s}}{\partial Y^{3}}=0$$

(22) $$Y=\eta ,\;\;\;\;\;\;\frac{\partial^{2}\theta_{f}}{\partial Y^{2}}=\frac{U_{p}\omega }{U_{m}\kappa }-\frac{BrU_{p}{}^{2}}{U_{m}{}^{2}\kappa }-\frac{BrDa}{U_{m}{}^{2}\epsilon \kappa }{\left(\frac{dU_{p}}{dY}\right)}^{2}=\varphi$$

(23) $$Y=\eta ,\;\;\;\;\;\;\frac{\partial^{2}\theta_{s}}{\partial Y^{2}}=0$$

where φ is a coefficient defined in Appendix A.

### 2.3. Analytical Solution

#### 2.3.1. Temperature

The analytical solution for the temperatures in clear fluid and porous medium are derived as follows:

(24) $$\theta_{c}=D_{1}Y+D_{2}Y^{2}+D_{3}Y^{3}+D_{4}Y^{4}+D_{5}$$

(25) $$\theta_{f}=E_{1}Y^{2}+E_{2}\cosh\left(A_{0}Y\right)+E_{3}\cosh\left(\sqrt{C_{0}}Y\right)+E_{4}\cosh\left(2A_{0}Y\right)+E_{5}$$

(26) $$\theta_{s}=E_{1}Y^{2}+F_{1}\cosh\left(A_{0}Y\right)+F_{2}\cosh\left(\sqrt{C_{0}}Y\right)+F_{3}\cosh\left(2A_{0}Y\right)+F_{4}$$

#### 2.3.2. Dimensional Temperature Variation with x and y

In the respective porous medium, and clear fluid region, the dimensional temperatures derived from Equations (24) and (25), are recast as

(27) $$T_{c}=\theta_{c}\frac{q_{w}H}{k_{s,eff}}+\frac{\partial T_{c}}{\partial x}x+T_{in}$$

(28) $$T_{f}=\theta_{f}\frac{q_{w}H}{k_{s,eff}}+\frac{\partial T_{f}}{\partial x}x+T_{in}$$

### 2.4. Problem Formulation

Nusselt number is defined as

(29) $$Nu=\frac{q_{w}H}{k_{f}\left(T_{w}-T_{m}\right)}$$

can be recast as

(30) $$Nu=\frac{2\epsilon }{\;\kappa (\theta_{w}-\theta_{m,f})}$$

where the bulk mean fluid temperature is obtained from

(31) $$\theta_{m,f}=\frac{1}{U_{m}}\left(\overset{\eta }{\underset{0}{\int }}U_{p}\theta_{f}dY+\overset{1}{\underset{\eta }{\int }}U_{c}\theta_{c}dY\right)$$

The new Nusselt number expression is hence obtained as

$$Nu=G_{1}+420\left(-2A_{2}D_{5}+A_{3}E_{2}+2DaE_{5}+12DaA_{0}{}^{3}E_{5}\right)\eta +\left(A_{3}E_{2}+2DaE_{4}\right)$$

$$3\sinh\left(2A_{0}\eta \right)+2A_{3}E_{4}\sinh\left(3A_{0}\eta \right)-420\left(A_{2}D_{1}+A_{1}D_{5}\right)\eta^{2}-140\;(2A_{1}D_{1}+2A_{2}D_{2}$$

$$-D_{5}-2DaE_{1}+4DaA_{0}{}^{3}E_{1})\eta^{3}+105\left[D_{1}-2\left(A_{1}D_{2}+A_{2}D_{3}\right)\right]\eta^{4}+84[D_{2}-2(A_{1}D_{3}$$

(32) $$+A_{2}D_{4})]\eta^{5}+70\left(D_{3}-2A_{1}D_{4}\right)\eta^{6}+60D_{4}\left(A_{3}E_{2}+2DaE_{4}\right)\eta^{7}$$

where the coefficient $G_{1}$ is given in Appendix A.

### 2.5. Verification of Results

Nusselt number is compared with the literature [40,41], reduced to cases completely filled with clear fluid $\left(\eta =0\right)$, and fully filled with porous medium (η=1). In the porous medium, as κ→∞,Bi→0, and Da→∞, a porous medium approaches a clear fluid [40]. Table 1 shows that the results in this study concur excellently with the literature.

## 3. Results and Discussion

The optimum volume fraction of porous medium η used is dependent on the parameters κ, Bi, and Da. For brevity, we fixed the thickness of the porous medium to be η=0.9, for all subsections except for Section 3.4, which used the optimum volume fraction η=0.45, in a microchannel.

### 3.1. Velcoity Profile

**Figure 2:**
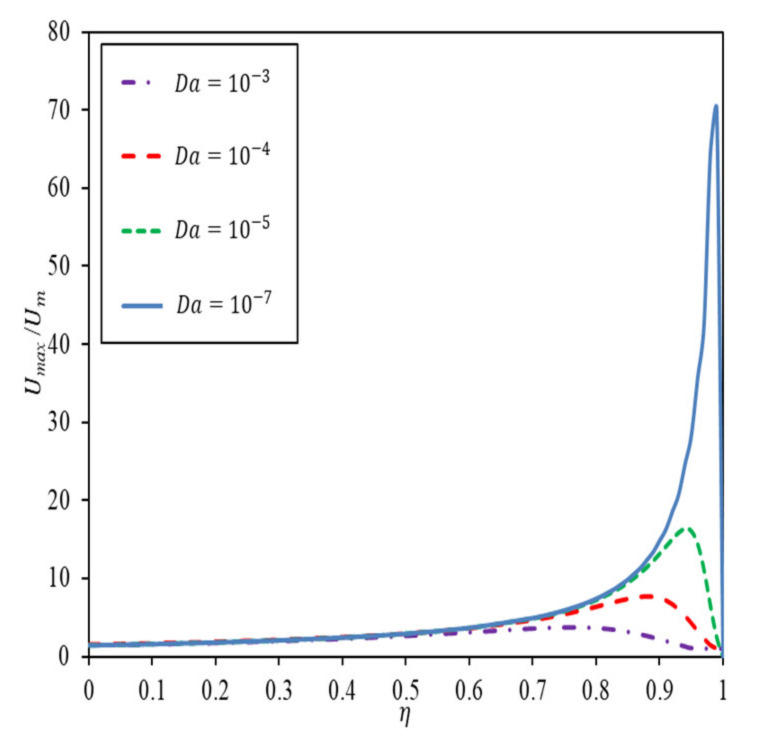
Maximum velocity achievable with porous medium thickness by having Da as a parameter.

Velocity profile presented in [24] is graphed to facilitate the discussion in the ensuing sections. Figure 2 represents the maximum velocity obtained across the channel for various Da and porous medium thickness. It depicts the highest velocity achievable for the respective Da, at a specific thickness of porous medium. Beyond this thickness, velocity drops as porous medium insert fills up the channel.

### 3.2. Temperature Profile

**Figure 3:**
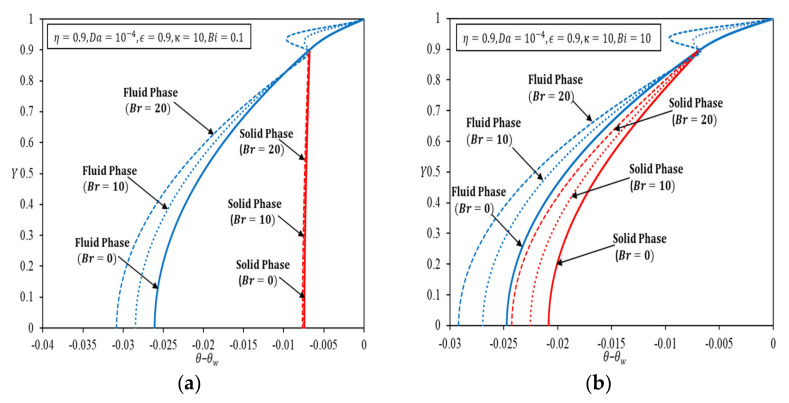

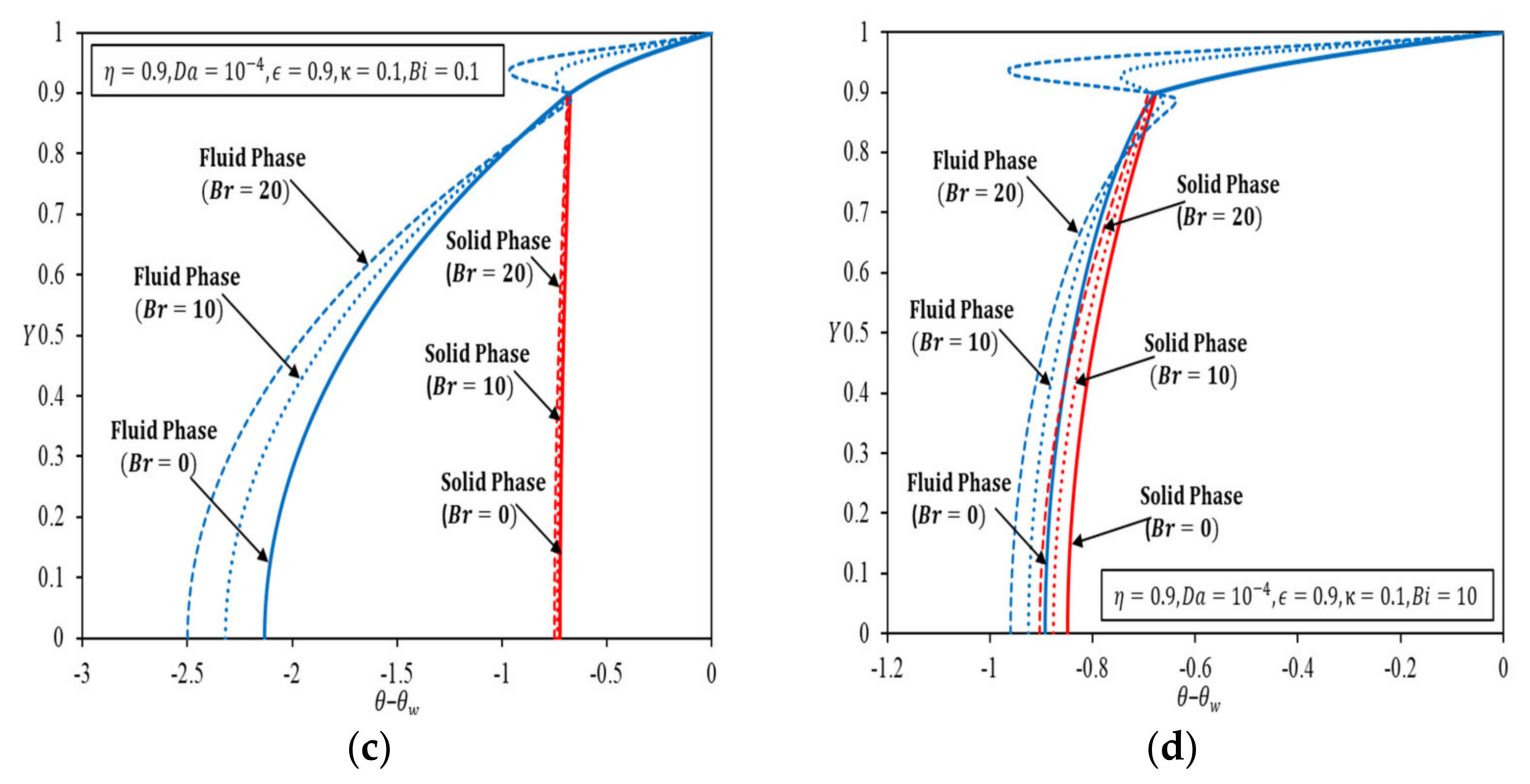
Temperature distributions for fluid and solid phases by having Br as a parameter for $Da=10^{-4}$ (**a**) κ=10, Bi=0.1; (**b**) κ=10, Bi=10; (**c**) κ=0.1, Bi=0.1; (**d**) κ=0.1, Bi=10.

Figure 3a–d depict the dimensionless temperature at specified Bi and κ for $Da=10^{-4}$, in a channel partially filled with porous medium up to η=0.9, with Br as a parameter. An increasing Br amplifies the temperature difference between solid and fluid in the porous region, as well as the clear fluid temperature. In Figure 3a, high *κ*, and low Bi render a large temperature difference between fluid and solid phases, indicating weak interfacial heat transfer between two phases. Fluid and solid temperatures are lowered as Br increases. For low κ and low Bi, the same trend is observed in Figure 3c, accompanied by a larger temperature drop due to viscous dissipation. When there is strong resistance to fluid conduction in porous medium, internal heat exchange from a higher solid temperature to a lower fluid temperature becomes significant; a small Bi gives rise to a large temperature difference. For a low κ and a big Bi, depicted in Figure 3d, heat flux bifurcation is observed near the interface with the oppositely signed temperature gradients in the fluid and solid phases. The phenomenon magnifies inside the porous medium as the viscous dissipation increases. Heat flux bifurcation is reinforced by dominant solid conduction when κ is small, and Bi is large.

Table 2 delineates the role of viscous dissipation in heat flux bifurcation at the interface region at fixed Br, for a set of stipulated parameters. It shows that increasing Br and Bi intensifies the phenomenon of heat flux bifurcation at the interface, while a decrease in Da further magnifies this phenomenon at the interface. As Da decreases, a more intense viscous dissipation resulting from a large velocity gradient in the interface region intensifies the heat flux bifurcation; manifested as the temperature fluctuation at the interface region. It is noteworthy that when the permeability of the porous medium deviates more from that of clear fluid, hence exerting larger resistances to the fluid and heat flow, heat flux bifurcation intensifies despite the fact that a composite channel with a lower Da gives a better overall convection heat transfer enhancement.

### 3.3. Nusselt Number

**Figure 4:**
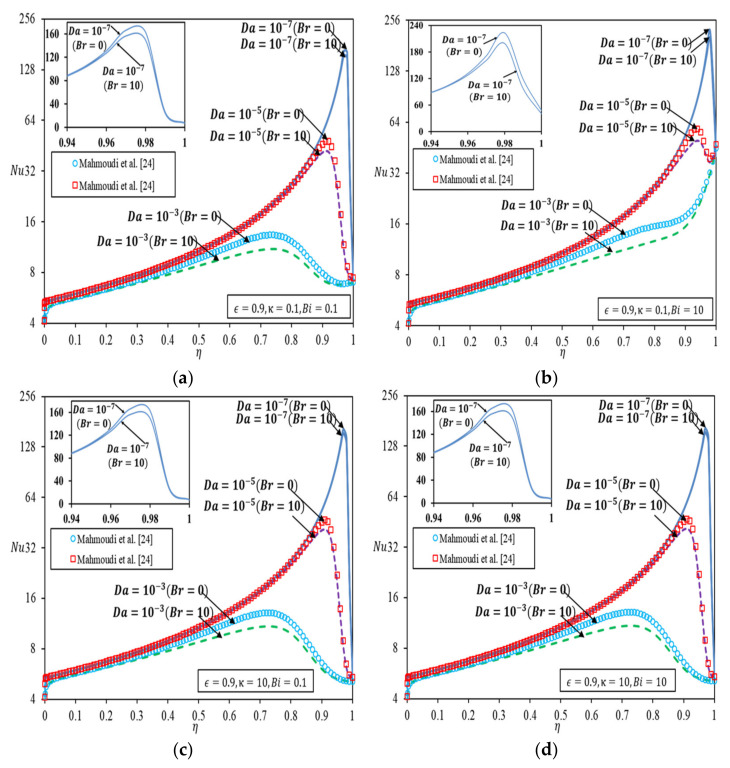
Nusselt number versus thickness of porous medium [24] (**a**) κ=0.1,Bi=0.1; (**b**) κ=0.1, Bi=10; (**c**) κ=10, Bi=0.1; (**d**) κ=10, Bi=10.

Figure 4 shows the Nu variation in the channel with the porous medium thickness, having Da and Br as parameters. The results are compared to those without viscous dissipation in Ref. [24].

In Figure 4a, for κ and Bi both specified as 0.1, Nu increases with an increase in the thickness of the porous medium and reaches a maximum at the optimal porous medium thickness. Beyond the optimal thickness, Nu drops more significantly as Da decreases. The surge in Nu is obvious as the thickness of porous medium constrained by the optimal thickness increases. The trend is not unexpected in light of the maximum velocity plot in Figure 2, whereby the optimal thickness corresponds to one that gives the highest achievable velocity. Nu decreases sharply toward a more packed porous medium due to a rather drastic drop in velocity, which ultimately reduces the heat transfer. The trend is alike in the presence of viscous dissipation, except for a reduced Nu. It is worth mentioning that the viscous dissipation effect is most prominent at the optimal porous medium thickness. Figure 4b depicts the Nu variation for κ=0.1 and Bi=10. The trend of variation for $Da=10^{-3}$ is different in this case, with the maximum Nu achieved for a fully filled channel (η=1). When κ is small, and Bi is large for a larger Da, the convection effect in a clear fluid is not sufficient to dissipate heat from the wall, hence extending the diffusion of heat to porous medium substantially. Under circumstances where the thermal resistance to fluid conduction is much larger than the thermal resistance to solid conduction, heat transfer in porous medium is dominated by a different transport mechanism as compared to that in Figure 4a, giving rise to an intensified heat flux bifurcation like what is shown in Figure 4d as heat tends to diffuse through the solid in porous medium. Nonetheless, viscous dissipation effects tend to resemble the velocity variation in Figure 2 for smaller Da, due to enhanced convection.

Likewise, for Figure 4c and d, as κ increases, the same trend is obtained, as discussed in Figure 4a. Figure 4c,d depict that increasing κ lowers the Nu, because less amount of heat flux is transferred to the solid phase in the porous region from the interface. The trend, however, is on the contrary to the results observed in [33], which demonstrated asymptotes in such a plot when the top wall is insulated while the bottom wall is heated when porous medium insert is placed next to a heated wall.

**Figure 5:**
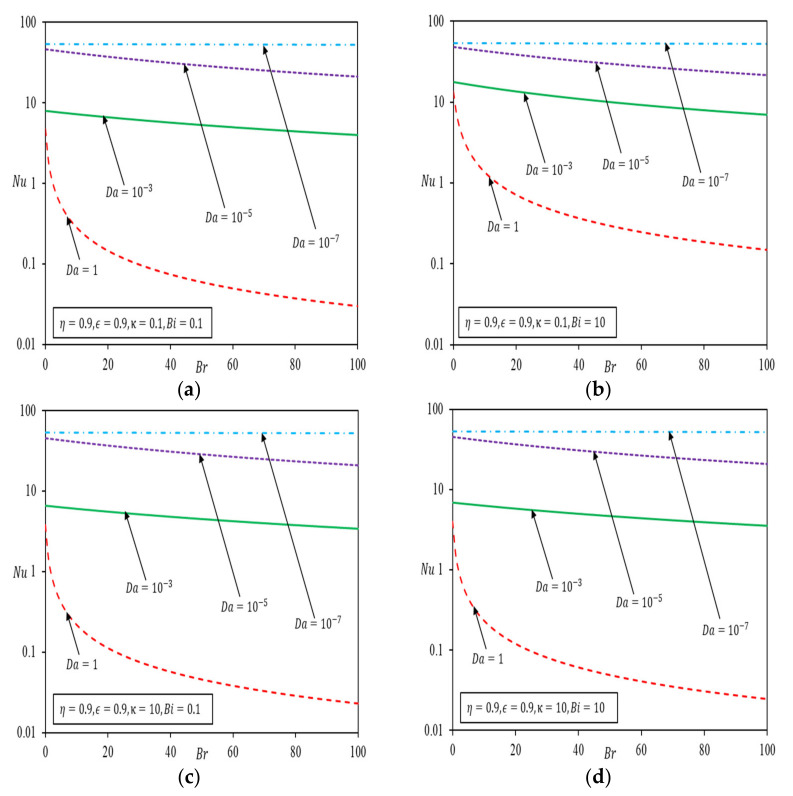
Nusselt number versus Brinkman number with porous medium thickness η=0.9. (**a**) κ=0.1, Bi=0.1 (**b**) κ=0.1, Bi=10 (**c**) κ=10, Bi=0.1 (**d**) κ=10, Bi=10.

Figure 5 depicts the variation of Nu against Br with Da as the parameter. Figure 5a,b are plotted for small κ values, with Bi fixed at 0.1 and 10, respectively, and Figure 5c,d are plotted for higher κ values for the same Bi. Figure 5a–d show the same pattern of variation whereby Nu decreases with increasing Br for all Da more significantly for larger Da. Nu decreases sharply toward a larger Da and an increasing Br due to a rather drastic hike in the velocity gradient, caused by viscous effects, which ultimately reduces the heat transfer. The extent of Br effect commensurate with the velocity surge at a specified porous medium filling as shown in Figure 2. Figure 5 also depicted that κ and Bi have a more obvious effect on Nu when these combined effects impose significant resistance to fluid conduction in porous region. Otherwise, Nu hinges largely on the convection effect inside the clear fluid only region.

### 3.4. Microchannel Partially Filled with Porous Medium

**Figure 6:**
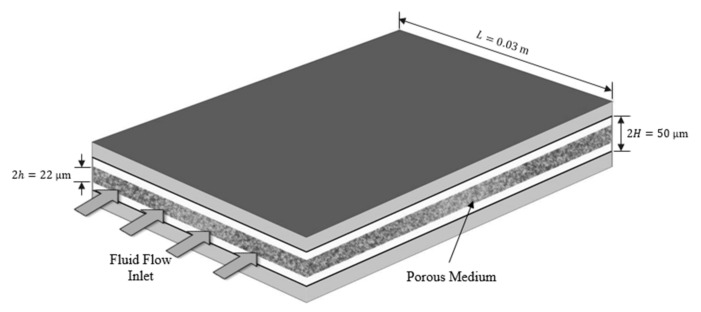
Schematic diagram of the composite microchannel.

The theoretical results in this study are applied to capture the effects of viscous dissipation in a microchannel partially filled with silicon-based porous medium saturated with Al_2_O_3_-Water nanofluid, as depicted in Figure 6. The specifications of the microchannel were taken from [42] and stated in Table 3.

The thermophysical properties of Al_2_O_3_ nanoparticles, obtained from the correlations in [43] with a $d_{np}$ of 40 nm are given in Table 4.

#### 3.4.1. Heat Transfer Coefficient

**Figure 7:**
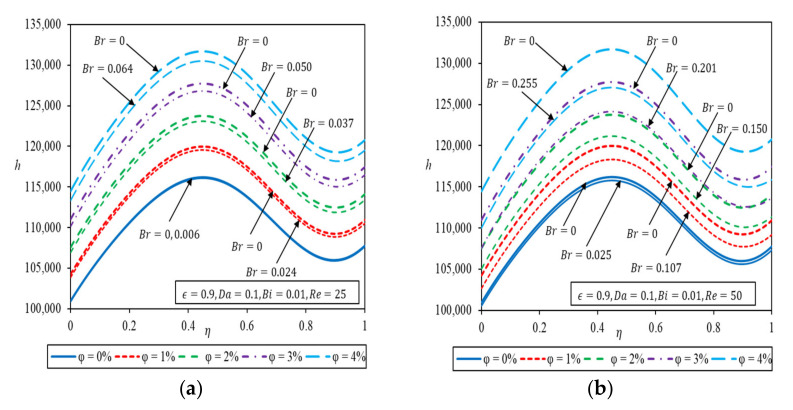
Heat transfer coefficient as a function of porous medium thickness with different volume fractions of Al_2_O_3_-Water nanofluid (**a**) Re=25 (**b**) Re=50.

The heat transfer coefficient for 0% to 4% volume fraction of Al_2_O_3_-Water nanofluid at Re=25 and 50 are presented in Figure 7a,b respectively with and without viscous dissipation. Figure 7 shows an increasing heat transfer coefficient with the volume fraction of nanoparticles. For this particular specification, the highest heat transfer coefficient is obtained when the microchannel is partially filled with a porous medium, η=0.45 due to the synergistic effect of a more uniform velocity distribution and temperature profile for Da=0.1. The heat transfer coefficients for water are lower than ones for nanofluid and are barely affected by viscous dissipation. As *Re* increases to Re=50 in Figure 7b, marked effect of viscous dissipation is notable where the heat transfer coefficient is lowered significantly because of the overriding effect of the heat source over heat convection.

#### 3.4.2. Temperature Field in a Microchannel

**Figure 8:**
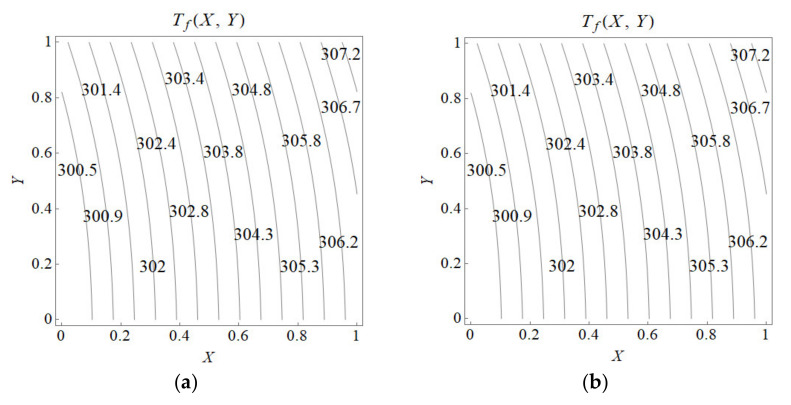

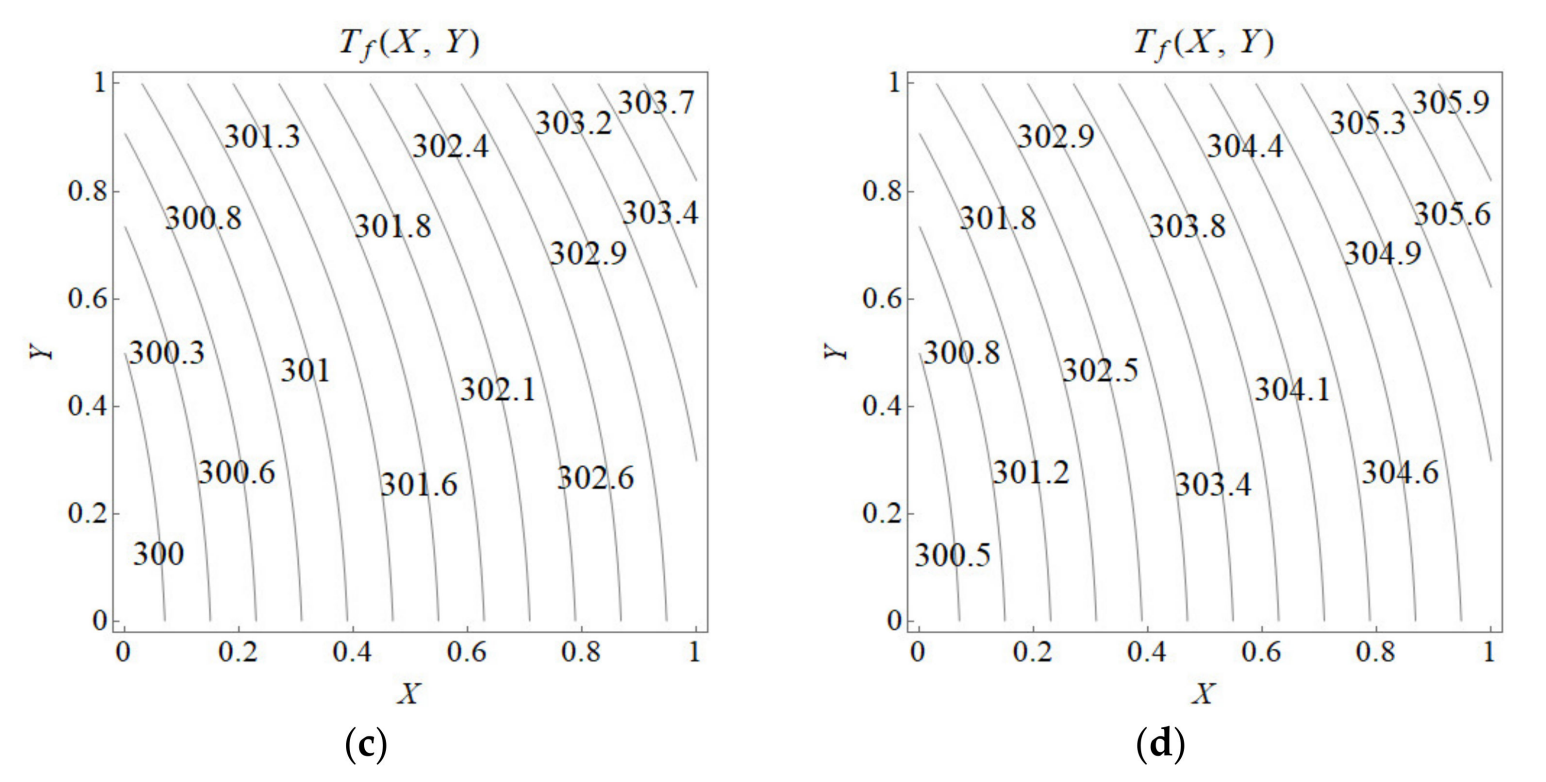
Temperature line plots in Kelvin for Re = 25 in a microchannel. (**a**) 0% volume fraction Al_2_O_3_-Water without viscous dissipation. (**b**) 0% volume fraction Al_2_O_3_-Water with viscous dissipation. (**c**) 4% volume fraction Al_2_O_3_-Water without viscous dissipation. (**d**) 4% volume fraction Al_2_O_3_-Water with viscous dissipation.

Two-dimensional temperature line plots are produced for a fluid phase in porous material and clear fluid region in the microchannel based on Equations (27) and (28) as depicted in Figure 8. Figure 8 is plotted for the same Da with a porous insert fraction, η=0.45, and the wall heat flux is specified as $1\times 10^{4}$ W/m^2^, the isotherms are calculated for a particular Reynolds number (Re), from which the mean flow velocity and Br are determined. A temperature of 300 K is assumed at X=0, the leading edge of lower wall. Figure 8a,b depicted the isotherms for 0% volume fraction of nanoparticles, whereas those of 4% volume fraction of nanoparticles were presented in Figure 8c,d for Re=25, indicating negligible influence of viscous dissipation. On the other hand, having Al_2_O_3_-Water nanofluid in Figure 8c,d, for the same Re, the fluid temperature elevates along the microchannel as the viscous dissipation effects are considered. Hence, nanofluid increases the temperature along the flow as viscous dissipation effect is considered. It is also noteworthy that the suspension of nanoparticles gives rise to a lower temperature when viscous dissipation is assumed negligible.

Table 5 compares the heat transfer coefficient for 0%, and 4% volume fraction of Al_2_O_3_-Water nanofluid at fixed Re for the partially filled porous medium, with η=0.45. Viscous dissipation causes a more significant deterioration in the heat transfer coefficient of the 4% volume fraction of Al_2_O_3_-Water nanofluid as compared to water at Re=25, and with an increased Re, Re=50, the heat transfer coefficient diminished by 6% in a composite microchannel having the 4% Al_2_O_3_-Water as coolant. Nanofluid is a better coolant than water but viscous dissipation is deemed to cause a more adverse effect on heat convection in a nanofluid flow as the Re and the corresponding *Br* go up in a microchannel.

## 4. Conclusions

New exact Nusselt numbers and temperature profiles in a channel partially filled with porous medium at the core are presented for a forced convective heat transfer, with viscous dissipation accounted for under local thermal non-equilibrium conditions. Viscous dissipation has the most significant effect on the temperature at the interface in cases where there is strong thermal resistance to fluid diffusion in porous material combined with strong hydrodynamics resistance to fluid flow, causing heat flux bifurcation at the interface. Viscous dissipation notably causes a more significant temperature decrease across the channel with an increasing *Da* and hence lowers the Nusselt number. The extent of Br effect commensurates with the highest velocity achievable at a specified porous medium filling, noting that convection in a clear fluid is not sufficient to dissipate the wall heat flux. The heat transfer performance deteriorates when the viscous dissipation effect is accounted for, more so when *Da* approaches 1, in line with the *Nu* trend. The two-dimensional isotherm plots in the microchannel highlighted the significance of viscous dissipation even for low Reynolds number flow. Maximum heat transfer coefficient is obtained with a certain fraction of porous medium in the channel. The 4% vol. fraction Al_2_O_3_-water is a better coolant than water in the microchannel in cases where the Reynolds number is low. It is noteworthy that viscous dissipation however has a more notable adverse impact on the heat transfer coefficient of the nanofluid, more so as Reynolds number increases. This study contributes to the heat transfer coefficient computation in a channel partially filled with a porous medium at the core, which is useful to forced convection in a partially filled microchannel where viscous dissipation is significant.

## Figures and Tables

**Table 1:** Nusselt number comparison with literature for LTNE model.

Thickness of Porous Medium, η	Brinkman Number, Br	Biot Number, Bi	Kappa, κ	Darcy Number, Da	Nu, Present Study	Nu [40]	Nu [41]
1	0	0.01	100	10	4.122	4.117	-
1	0	1	0.01	1	123.9	-	123.9
1	0	1	100	$10^{-4}$	5.901	-	5.901
1	0	1	100	1	4.171	-	4.171
1	1	0.01	100	10	0.2444	0.2439	-
1	1	10	0.01	1	132.4	-	132.4
1	1	1	0.01	1	49.33	-	49.33
1	1	1	100	1	1.622	-	1.622
1	1	1	0.01	0.01	143.3	-	143.3
1	10	0.01	100	10	0.0258	0.0257	-
1	10	1	0.01	0.01	122.6	-	122.6
1	10	1	0.01	0.04	85.37	-	85.37

**Table 2:** Heat flux bifurcation for fluid and solid phases in porous region at interface η=0.9.

	Br	$Da=10^{-3}$		$Da=10^{-4}$	
		$\theta_{f}{}^{’}$	$\theta_{s}{}^{’}$	$\theta_{f}{}^{’}$	$\theta_{s}{}^{’}$
κ=0.1, Bi=0.1	10	1.6450	0.16855	−3.3145	0.09494
	20	−3.43828	0.17674	−10.326	0.10406
κ=0.1, Bi=10	10	−2.9101	0.62406	−5.9737	0.36085
	20	−7.5618	0.58943	−12.987	0.37010

**Table 3:** Specifications of a microchannel [42].

Nanofluid	Al_2_O_3_-Water
Solid	Silicon
Length of the channel, L(m)	0.03
Channel height, $2H\;\left(\mu m\right)$	50
Heat flux, $q_{w}$ (W/m^2^)	1 ×10^4^
Thermal conductivity of solid, $k_{s}$(W/m·K)	148
Porosity, ϵ	0.9
Darcy number, Da	0.1
Biot number, Bi	0.01

**Table 4:** Thermophysical properties of nanofluid [42].

Properties	Nanoparticle (Al_2_O_3_)	Base Fluid (Water)
ρ (kg/m^3^)	3975	997
$c_{p}$ (J/kg·K)	778.6	4179
k (W/m·K)	36	0.613
μ (N·s/m^2^)	-	8.55×10^−4^

**Table 5:** Comparison of heat transfer coefficient for a composite channel.

Working Fluid	Re	Br	h$,\;W/m^{2}\cdot ℃$ at η=0.45	% Decrease in h
Water	25	0	116,191	0.10
		0.006	116,086	
4% volume fraction Al_2_O_3_-Water nanofluid		0	132,800	2.44
		0.064	129,550	
Water	50	0	116,191	0.36
		0.025	115,773	
4% volume fraction Al_2_O_3_-Water nanofluid		0	132,800	5.62
		0.255	125,054	

## Data Availability

Not applicable.

## Appendix A

$$\varphi =\frac{\omega U_{m}\left[Da+A_{3}\cosh\left(A_{0}\eta \right)\right]-Br{\left[Da+A_{3}\cosh\left(A_{0}\eta \right)\right]}^{2}-A_{0}{}^{2}A_{3}{}^{2}BrDa\sinh{\left(A_{0}\eta \right)}^{2}/\epsilon }{\kappa U_{m}{}^{2}}$$

$$A_{0}=\sqrt{\epsilon /Da}$$

$$A_{1}=\frac{2\sqrt{Da}\eta \cosh\left(A_{0}\eta \right)-\left(-1+2Da+\eta^{2}\right)\epsilon^{3/2}\sin h\left(A_{0}\eta \right)}{2\sqrt{Da}\eta \cosh\left(A_{0}\eta \right)-2\left(-1+\eta \right)\epsilon^{3/2}\sin h\left(A_{0}\eta \right)}$$

$$A_{2}=\frac{\sqrt{Da}\left(1-2\eta \right)\cosh\left(A_{0}\eta \right)+\left[2Da+\left(-1+\eta \right)\eta \right]\epsilon^{3/2}\sin h\left(A_{0}\eta \right)}{2\sqrt{Da}\eta \cosh\left(A_{0}\eta \right)-2\left(-1+\eta \right)\epsilon^{3/2}\sin h\left(A_{0}\eta \right)}$$

$$A_{3}=\frac{\sqrt{Da}\left[-2Da+{\left(-1+\eta \right)}^{2}\right]}{2\sqrt{Da}\cosh\left(A_{0}\eta \right)-2\left(-1+\eta \right)\epsilon^{3/2}\sin h\left(A_{0}\eta \right)}$$

$$C_{0}=\frac{Bi\left(1+\kappa \right)}{\kappa }$$

$$D_{1}=\frac{\left[2\left(1-3A_{1}+3A_{1}{}^{2}\right)BrDa+U_{m}\left(\omega -3A_{1}\omega -6A_{2}\omega +6U_{m}\right)\right]\epsilon }{6\kappa U_{m}{}^{2}}$$

$$D_{2}=\frac{\left(-A_{1}{}^{2}BrDa+A_{2}\omega U_{m}\right)\epsilon }{2\kappa U_{m}{}^{2}}$$

$$D_{3}=\frac{A_{1}\left(2BrDa+\omega U_{m}\right)\epsilon }{6\kappa U_{m}{}^{2}}$$

$$D_{4}=\frac{-\left(2BrDa+\omega U_{m}\right)\epsilon }{24\kappa U_{m}{}^{2}}$$

$$D_{5}=\frac{\eta }{24\kappa U_{m}{}^{2}}\{2BrDa\left[-4+6A_{1}{}^{2}\left(-2+\eta \right)+\eta^{3}-4A_{1}\left(-3+\eta^{2}\right)\right]+\{\omega [-4$$

$$-12A_{2}\left(-212A_{2}\left(-2+\eta \right)+\eta^{3}-4A_{1}\left(-3+\eta^{2}\right)\right]-24U_{m}\}U_{m}\}\epsilon$$

$$E_{1}=\frac{Bi\left\{A_{0}{}^{2}A_{3}{}^{2}BrDa-\left[A_{3}{}^{2}Br+2Da\left(BrDa-\omega U_{m}\right)\right]\epsilon \right\}}{4C_{0}\kappa \epsilon U_{m}{}^{2}}$$

$$E_{2}=-\frac{A_{3}\left(A_{0}{}^{2}-Bi\right)\left(2BrDa-\omega U_{m}\right)}{\kappa U_{m}{}^{2}\left(A_{0}{}^{4}-A_{0}{}^{2}C_{0}\right)}$$

$$E_{3}=\frac{1}{2C_{0}{}^{2}(4A_{0}{}^{4}-5A_{0}{}^{2}C_{0}+C_{0}{}^{2})\kappa^{2}U_{m}{}^{4}\epsilon }\{\left(4A_{0}{}^{2}-C_{0}\right)C_{0}A_{3}\left(A_{0}{}^{2}-Bi\right)(2BrDa$$

$$-\omega U_{m})\cosh\left(A_{0}\eta \right)+\left(A_{0}{}^{2}-C_{0}\right)[\left(4A_{0}{}^{2}-C_{0}\right)[2\kappa \epsilon U_{m}{}^{2}A_{6}\varphi -A_{3}{}^{2}\left(4A_{0}{}^{2}-Bi\right)Br\left(A_{0}{}^{2}Da+\epsilon \right)$$

$$+C_{0}C_{2}\cosh\left(2A_{6}\eta \right)]\}\sec h\left(C_{0}\eta \right)\}$$

$$E_{4}=-\frac{A_{3}{}^{2}\left(4A_{0}{}^{2}-Bi\right)Br\left(A_{0}{}^{2}Da+\epsilon \right)}{2\kappa \epsilon U_{m}{}^{2}\left(16A_{0}{}^{4}-4A_{0}{}^{2}C_{0}\right)}$$

$$E_{5}=-\frac{1}{16\kappa^{2}\epsilon^{2}U_{m}{}^{2}A_{0}{}^{2}C_{0}{}^{2}}\{2A_{0}{}^{2}[4\kappa \epsilon U_{m}{}^{2}C_{0}\varphi -2Bi\{A_{0}{}^{2}A_{3}{}^{2}BrDa-[A_{3}{}^{2}Br$$

$$+2Da\left(BrDa-\omega U_{m}\right)]\epsilon \}+C_{0}\eta^{2}Bi\{A_{0}{}^{2}A_{3}{}^{2}BrDa-\left[A_{3}{}^{2}Br+2Da\left(BrDa-\omega U_{m}\right)\right]\epsilon ]\}$$

$$+4C_{0}A_{3}\left(A_{0}{}^{2}-Bi\right)\left(2BrDa-\omega U_{m}\right)\cosh\left(A_{0}\eta \right)+C_{0}A_{3}\left(A_{0}{}^{2}-Bi\right)\left(2BrDa-\omega U_{m}\right)$$

$$\cosh\left(2A_{0}\eta \right)\}$$

$$F_{1}=-\frac{A_{3}Bi\left(-2BrDa+\omega U_{m}\right)}{\kappa U_{m}{}^{2}\left(A_{0}{}^{4}-A_{0}{}^{2}C_{0}\right)}$$

$$F_{2}=\frac{1}{2\kappa^{2}\epsilon U_{m}{}^{4}C_{0}{}^{2}(4A_{0}{}^{2}-5A_{0}{}^{2}C_{0}+C_{0}{}^{2})}\{-\left(4A_{0}{}^{2}-C_{0}\right)C_{0}A_{3}Bi\left(-2BrDa+\omega U_{m}\right)$$

$$\cosh\left(A_{0}\eta \right)+\left(A_{0}{}^{2}-C_{0}\right)[4A_{0}{}^{2}Bi\left\{A_{0}{}^{2}A_{3}{}^{2}BrDa-\left[A_{3}{}^{2}Br+2Da\left(BrDa-\omega U_{m}\right)\right]\epsilon \right\}$$

$$-C_{0}Bi\left\{A_{0}{}^{2}A_{3}{}^{2}BrDa-\left[A_{3}{}^{2}Br+2Da\left(BrDa-\omega U_{m}\right)\right]\epsilon \right\}+C_{0}A_{3}{}^{2}BiBr\left(A_{0}{}^{2}Da+\epsilon \right)$$

$$\cosh\left(2A_{0}\eta \right)\}\sec h\left(C_{0}\eta \right)\kappa U_{m}{}^{2}\}$$

$$F_{3}=\frac{A_{3}{}^{2}BiBr\left(A_{0}{}^{2}Da+\epsilon \right)}{2\kappa \epsilon U_{m}{}^{2}\left(16A_{0}{}^{4}-4A_{0}{}^{2}C_{0}\right)}$$

$$F_{4}=-\frac{2A_{0}{}^{2}\left(-2+C_{0}\eta^{2}\right)+4C_{0}A_{3}Bi\left(-2BrDa+\omega U_{m}\right)\cosh\left(A_{0}\eta \right)+C_{0}\cosh\left(A_{0}\eta \right)}{4A_{0}{}^{2}C_{0}{}^{2}}$$

$$G_{1}=\left(2\epsilon \right)/\{\kappa \{\left(D_{1}+D_{2}+D_{3}+D_{4}+D_{5}\right)-\frac{1}{U_{m}}\{\frac{1}{840}[-2\left(42D_{2}+35D_{3}+30D_{4}+70D_{5}\right)$$

$$+7[5\left(-3+8A_{1}+12A_{2}\right)D_{1}+2A_{2}\left(20D_{2}+15D_{3}+12D_{4}+60D_{5}\right)+A_{1}(30D_{2}+24D_{3}+20D_{4}$$

$$+60D_{5})]+1/\left(A_{0}{}^{2}-C_{0}\right)[A_{0}A_{3}\cosh\left(\sqrt{C_{0}}\eta \right)\sinh\left(A_{0}\eta \right)]+\frac{1}{12A_{0}{}^{3}}[-24A_{0}A_{3}E_{1}\eta \cosh\left(A_{0}\eta \right)$$

$$+\left(12A_{0}{}^{2}DaE_{2}+6A_{0}{}^{2}A_{3}E_{4}+126A_{0}{}^{2}A_{3}E_{5}+24A_{3}E_{1}+12A_{0}{}^{2}\eta^{2}\right)]\sinh\left(A_{0}\eta \right)+6A_{0}{}^{3}A_{3}E_{2}\eta$$

$$+1/\sqrt{C_{0}}\left(A_{6}-A_{0}{}^{2}\right)E_{3}\sinh\left(\sqrt{C_{0}}\eta \right)[\left(A_{6}-A_{0}{}^{2}\right)DaA_{3}A_{6}\cosh\left(A_{0}\eta \right)]$$
